# What do we know about the global methane budget? Results from four decades of atmospheric CH_4_ observations and the way forward

**DOI:** 10.1098/rsta.2020.0440

**Published:** 2021-11-15

**Authors:** Xin Lan, Euan G. Nisbet, Edward J. Dlugokencky, Sylvia E. Michel

**Affiliations:** ^1^ US National Oceanic and Atmospheric Administration, Global Monitoring Laboratory, 325 Broadway, Boulder, CO 80305 USA; ^2^ Cooperative Institute for Research in Environmental Sciences, University of Colorado, Boulder, CO 80309, USA; ^3^ Department of Earth Sciences, Royal Holloway, University of London, Egham, Surrey TW20 0EX, UK; ^4^ Institute of Arctic and Alpine Research, University of Colorado, Boulder, CO, USA

**Keywords:** methane, *in situ* observations, time series

## Abstract

Atmospheric CH_4_ is arguably the most interesting of the anthropogenically influenced, long-lived greenhouse gases. It has a diverse suite of sources, each presenting its own challenges in quantifying emissions, and while its main sink, atmospheric oxidation initiated by reaction with hydroxyl radical (OH), is well-known, determining the magnitude and trend in this and other smaller sinks remains challenging. Here, we provide an overview of the state of knowledge of the dynamic atmospheric CH_4_ budget of sources and sinks determined from measurements of CH_4_ and *δ*^13^C_CH4_ in air samples collected predominantly at background air sampling sites. While nearly four decades of direct measurements provide a strong foundation of understanding, large uncertainties in some aspects of the global CH_4_ budget still remain. More complete understanding of the global CH_4_ budget requires significantly more observations, not just of CH_4_ itself, but other parameters to better constrain key, but still uncertain, processes like wetlands and sinks.

This article is part of a discussion meeting issue 'Rising methane: is warming feeding warming? (part 1)'.

## 1. Introduction

Atmospheric CH_4_ absorbs terrestrial infrared radiation in a band at approximately 7.6 µm, so it affects Earth's radiation balance and is a primary driver of the human impact on climate. Based on measurements of air extracted from ice cores and firn, we know that atmospheric CH_4_ increased from approximately 360 ppb (see note about units in electronic supplementary material) during glacial periods to approximately 700 ppb during interglacial periods [1] as receding high-northern latitude glacial ice and changes to monsoon circulation resulted in more wetland area. This change was initiated by small variations in Earth's orbital parameters that affected the seasonal distribution of solar radiation across Earth's surface, but most of the increase in atmospheric CH_4_ likely resulted from feedbacks in the carbon cycle [2]. Natural emissions in the southern tropics may have driven a sustained atmospheric CH_4_ increase starting 5000 years before present [3]. These Holocene and earlier paleo records demonstrate the strong sensitivity of atmospheric CH_4_ to changing climate.

By contrast, the atmospheric CH_4_ growth from pre-industrial (approx. 720 ppb) to 2020 (1879.2 ± 0.6 ppb) was predominantly caused by human activity; it results in 0.52 W m^−2^ additional direct heating (see: e.g. https://www.esrl.noaa.gov/gmd/ccgg/ghgpower/, references therein, and electronic supplementary material), behind only CO_2_ (with 2.13 W m^−2^). There is an additional approximately 0.3 W m^−2^ from indirect effects of CH_4_'s atmospheric chemistry [4], specifically the production of tropospheric O_3_ and stratospheric H_2_O.

Developing sensible mitigation strategies for methane [5] requires a quantitative understanding of individual source and sink terms. Strong climate feedbacks similar to those that drove changes in atmospheric CH_4_ abundance in the Holocene have the potential to greatly enhance CH_4_ emissions, both in the Arctic, where current warming might release huge stores of carbon (greater than 1 Pg C in the top 3 m of permafrost [6]) as CH_4_ or CO_2_, depending on hydrology, and also in the tropics, where wetlands, including flooded land used for rice agriculture, and cattle may respond to warmth and rainfall.

## 2. Observations of atmospheric CH_4_ abundance and *δ*^13^C_CH4_ as budget constraints

### (a) Experimental methods

Blake *et al*. [7] showed that CH_4_ in the surface atmosphere varied spatially and seasonally across the globe. Recognizing the importance of improved understanding of the global CH_4_ budget for climate, NOAA began measurements of atmospheric CH_4_ from discrete air samples collected in its existing Cooperative Global Air Sampling Network in 1983, expanding available high-quality data both in frequency and spatially [8] to better constrain the global CH_4_ budget. Since 1998, *δ*^13^C_CH4_ has been measured in a subset of the same air samples by the Institute of Arctic and Alpine Research (INSTAAR), University of Colorado [9]. Currently, many laboratories around the world monitor atmospheric CH_4_ abundance and a few measure *δ*^13^C_CH4_. To help ensure the availability of comparable, high-quality observations for global CH_4_ budget studies, these efforts are organized under the umbrella of the World Meteorological Organization (WMO) Global Atmosphere Watch (GAW) program, and data are reported to the World Data Center for Greenhouse Gases hosted by the Japan Meteorological Agency (https://gaw.kishou.go.jp/). Even with this structure in place, there are still ‘ease of use’ issues; not all laboratories report data in a timely fashion, they report data on different standard scales without providing conversion to the WMO GAW CH_4_ mole fraction scale, uncertainties are not reported, etc. Isotopic data are further hindered by a lack of CH_4_-in-air reference materials and different methods of tracing *δ*^13^C_CH4_ to calcite reference materials [10]. To circumvent these issues, some research programs have collected data and made packages of observations available (see e.g. https://www.esrl.noaa.gov/gmd/ccgg/obspack/). Future effort is required to ensure high-quality measurements can be traced back to common standard scales, because our understanding of the global CH_4_ budget relies on precise spatio-temporal differences from these measurements. The data should also be publicly accessible in a timely manner, fully quality controlled and ready for scientific analysis; data users should not have to add uncertainties nor ensure traceability to a common standard scale.

We do not cover column CH_4_ abundances retrieved from measurements of radiance by satellite or surface IR sensors, which are not direct, calibrated measurements of CH_4_ abundance and have potential biases similar in magnitude to the large-scale signals we are trying to determine. While they are still useful, especially in the tropics, where direct measurements remain sparse, and in regional studies, they will always require calibrated direct measurements of CH_4_ for evaluation.

Here, we focus on NOAA CH_4_ and INSTAAR *δ*^13^C_CH4_ measurements in air samples collected from the Global Monitoring Laboratory's Cooperative Global Air Sampling Network. The network and sampling procedures were described in Dlugokencky *et al*. [11]. Briefly, weekly discrete whole-air samples are collected in pairs of borosilicate glass flasks with PTFE O-ring seals using a portable air sampler from a globally distributed network of air sampling sites (https://www.esrl.noaa.gov/gmd/ccgg/flask.html). Air samples were analysed for CH_4_ by gas chromatography with flame ionization detection through mid-2019. Since then, samples have been analysed for CH_4_ using a cavity ring-down spectrometer (CRDS). Responses of the GC/FID and CRDS were calibrated for CH_4_ abundance against a CH_4_ standard scale maintained at NOAA ([12]; this scale serves as the WMO CH_4_ mole fraction scale, X2004A). These data provide a spatially and temporally consistent set of observations. INSTAAR began analysing a subset of air samples for *δ*^13^C_CH4_ in 1998. CH_4_ from the sample is pre-concentrated, separated from other remaining components in a GC, combusted to CO_2_ and the CH_4_-derived CO_2_ analysed by continuous flow isotope ratio mass spectrometry. CH_4_ and *δ*^13^C_CH4_ data are available from NOAA's data server: ftp://aftp.cmdl.noaa.gov/data/trace_gases/xxx/flask/, where xxx = ‘ch4’ or ‘ch4c13’.

### (b) Documenting CH_4_'s global burden

**Figure 1.**
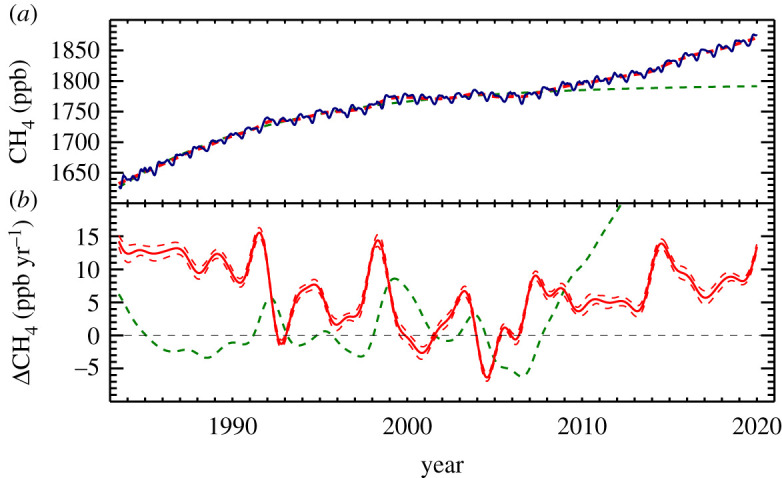
(*a*) Globally averaged atmospheric CH_4_ at Earth's surface at weekly resolution (blue). The red line is a deseasonalized trend fitted to the data. The green dashed line is the result of fitting equation (2.3) to the global means from 1983 through 2006. (*b*) Time derivative of the trend in (*a*) (solid red line). The green dashed curve is the difference, in ppb, between the trend and fit to equation (2.3). It represents anomalies from the steady-state model. (Online version in colour.)

Observations of the spatial and temporal distribution of atmospheric CH_4_ abundance provide key constraints on the global CH_4_ budget. In 2020, preliminary globally averaged CH_4_ determined by smoothing data as described below was 1879.2 ± 0.6 ppb, with an increase of 14.8 ± 0.5 ppb from January 1, 2020 to January 1, 2021 (https://gml.noaa.gov/ccgg/trends_ch4/; downloaded 8 July 2021). This implies an imbalance between emissions and sinks in 2020 of 41.0 Tg CH_4_ (based on 1 globally averaged ppb = 2.77 Tg CH_4_ [13]). The time series of these observations from 1983.5 through 2019 is shown in figure 1*a*. Global (and zonal) means are calculated by first smoothing data from a subset of sampling sites that capture well-mixed, background air, extracting values from the smoothed curve fits at synchronized time steps, then smoothing again as a function of latitude to define a matrix of CH_4_ abundance as a function of time (48 values per year) and latitude (intervals of sine (latitude) = 0.05). The quality of the data ensures that the variations in growth rate are significant, as indicated by the uncertainties (dashed lines in figure 1*b*) determined with a combination of resampling of the network (bootstrap; [14]) and Monte Carlo (to assess measurement uncertainty) methods (see electronic supplemenatry material for details).

### (c) Long-term changes

From the start of NOAA's measurements in 1983 through 2006, the CH_4_ growth rate was decreasing with significant interannual variability (IAV) superimposed on top of that. Then, globally averaged CH_4_ began increasing again in 2007.

For 1983–2006, if we assume no trend in the atmospheric CH_4_ lifetime, then the atmospheric CH_4_ burden was approaching steady state [15]. Starting from a global mass balance and rearranging to solve for emissions we have:

2.1 $$E=\frac{\text{d}[\text{C}{\text{H}}_{4}]}{\text{d}t}+\frac{\left[{\mathrm{CH}}_{4}\right]}{\tau },$$

where *E* is emissions, [CH_4_] is the global annual mean CH_4_ burden and d[CH_4_]/d*t* its annual increase, both determined from our surface global means, and *τ* is the atmospheric CH_4_ lifetime. After calculating emissions using *τ* = 9.1 yr [16] and fitting a straight line to emissions from 1984 through 2006, we find no trend in emissions (electronic supplementary material, figure S1; slope = 0.0 ± 0.3 Tg CH_4_ yr^−1^; uncertainty is 68% C.I.). It may seem surprising that atmospheric CH_4_ continues to increase while emissions and lifetime are constant, but this is expected from a chemical system with pseudo-first-order loss approaching steady state,

2.2 $${\left[\text{C}{\text{H}}_{4}\right]}_{t}={\left[\text{C}{\text{H}}_{4}\right]}_{ss}(1-\text{exp}[-t/\tau ]).$$

Accounting for the atmospheric CH_4_ abundance at the start of our measurements gives

2.3 $${\left[\text{C}{\text{H}}_{4}\right]}_{t}={\left[\text{C}{\text{H}}_{4}\right]}_{ss}-({\left[\text{C}{\text{H}}_{4}\right]}_{ss}-{\left[\text{C}{\text{H}}_{4}\right]}_{0})\text{exp}[-t/\tau ]),$$

where subscripts ‘ss’ and ‘0’ are for steady state and initial conditions (start of measurements). Equation (2.3) is fitted to the global means from 1983.5 through 2006, solving for [CH_4_]_ss_, ([CH_4_]_ss_ - [CH_4_]_0_), and *τ* to give a steady-state value of 1794 ppb and a lifetime of 9.2 yr, in agreement with IPCC's Fifth Assessment Report CH_4_ budget lifetime of 9.1 yr [16]. A curve calculated using the coefficients of the fit, extrapolated to 2020, is plotted in figure 1*a* (green), and the difference between it and the deseasonalized trend (green, in ppb) is shown with the growth rate (red) in figure 1*b*.

Since 2007, when this difference starts increasing dramatically, there has been a significant change in the global CH_4_ budget that will be explored later. Our conclusion of no trend in emissions from 1983 to 2006 is true only if there has been no trend in *τ*_CH4_, which is the largest source of uncertainty in this simple global mass balance. For changes in global CH_4_ emission rates to be consistent with the observed global means, *τ*_CH4_ would have had to change in a way that still coincidentally fits this steady-state model.

### (d) Interannual variability

In addition to the long-term changes in atmospheric CH_4_, IAV is also evident in the growth rate and the residuals (figure 1*b*), and it has inherent information about budget processes. When the process, or processes, responsible for IAV can be identified, it allows us to quantitatively test our understanding. An example is a large increase in CH_4_ growth rate in 1991 (red line in figure 1*b*) and the positive anomaly in the difference between the trend and steady-state fit in 1991 (green line in figure 1*b*). In this smoothed representation, which is similar to a 12-month running mean, the anomaly peaked at approximately 5 ppb; at weekly time resolution, it was approximately 9 ppb (see electronic supplementary material, figure S2). The subsequent decrease in 1992 growth rate is a result of the trend adjusting back to the long-term behaviour as indicated by the difference between the trend and steady-state fit returning to near-zero (green line in figure 1*b*).

Dlugokencky *et al*. [17] attributed this positive anomaly to the impact of the eruption of Mt. Pinatubo on the atmospheric CH_4_ sink. SO_2_ and ash from the eruption, and subsequent sulfate aerosol produced from the oxidation of SO_2_, decreased transmission of UV radiation, which decreased production of O(^1^D) and, ultimately, primary production of hydroxyl radical (OH). Qualitatively, this made sense, since anomalies in CO abundance were coincident and consistent in magnitude with the changes in CH_4_. Bândă *et al*. [18] investigated this change with a three-dimensional global chemistry model and found SO_2_ and sulfate reduced the tropospheric CH_4_ sink from reaction with OH by 17.8 Tg CH_4_ from June, 1991 to June, 1993, which only explained approximately 40% of the change in atmospheric CH_4_ burden from June, 1991 to May, 1992. Bândă *et al*. [19] identified additional factors that contributed significantly to changes in atmospheric CH_4_ growth rate including: changes in stratospheric O_3_, which also affected UV flux; atmospheric temperature, which affected CH_4_ emission rates from wetlands and the rate of oxidation of CH_4_ (k_OH+CH4_ is strongly temperature-dependent, approximately 2% K^−1^); biomass burning; and changes in rates of emission of other compounds that affect OH concentration (e.g. CO and NMHCs).

### (e) Spatial patterns as budget constraints

Because atmospheric mixing is not instantaneous, signals of sources and sinks exist in the atmospheric CH_4_ distribution that are useful for quantifying emissions. Fung *et al*. [13] compared the time-averaged latitude gradient calculated with a chemical transport model (CTM) to NOAA CH_4_ observations (electronic supplementary material, figure S3) to rule out specific emission scenarios that were inconsistent with atmospheric observations. They showed, for example, that early estimates of CH_4_ emissions from northern wetlands were too large to be consistent with the observed latitude gradient in surface CH_4_.

We exploited the gradient further by analysing temporal changes in spatial patterns to identify changes in the distribution of emissions, although this analysis cannot quantitatively determine the magnitude of the emission changes. Following the economic collapse in the former Soviet Union, Dlugokencky *et al*. [20] showed that a change in inter-polar CH_4_ difference (IPD; the difference between zonal averages calculated for 53–90° N and 53–90° S) was consistent with a decrease in CH_4_ emissions from the former Soviet Union of approximately 10 Tg between 1991 and 1992. Agreement between observations and a forward simulation with an atmospheric transport model (TM3) using emission estimates from Emissions Database for Global Atmospheric Research (EDGAR) was quite good; both showed a decrease of approximately 12 ppb in annual means calculated for high-northern latitude sites relative to the South Pole between the mid-1980s and the late-1990s (electronic supplementary material, figure S4).

Such analyses are only qualitative, because observed trends in spatial patterns are affected by changes in atmospheric transport, not just emissions [21], so quantitative estimates of emission changes must be assessed with a CTM. However, even without the rigour of a transport model, the observations show that, as of 2019, IPD has not recovered to the levels seen in the late-1980s (see electronic supplementary material, figure S5), suggesting there have not been large increases in Arctic CH_4_ emissions from clathrates and permafrost carbon in response to increasing temperature.

### (f) Other tracers: *δ*^13^C_CH4_

We get additional information about processes responsible for emitting CH_4_ from other tracers. For example, anomalies in CO abundance measured in the same air samples measured for CH_4_ were the right magnitude to be consistent with a decreased atmospheric OH sink after the eruption of Mt. Pinatubo. CO is also used as a tracer for biomass burning, where its molar emission ratio with CH_4_ is significantly larger than for the sink process. Ethane (C_2_H_6_) has been used as a tracer for fossil fuel emissions of CH_4_. Both are also emitted from other sources, which can complicate their interpretation. For example, Lan *et al*. [22] analysed long-term observations of CH_4_ and a suite of NMHCs from samples collected at a site significantly influenced by oil and gas production. Because emission ratios of C_2_H_6_/CH_4_ from fossil fuel production vary with time, predicting trends in CH_4_ enhancements from trends in C_2_H_6_ (and other NMHCs) enhancements and an assumed constant C_2_H_6_/CH_4_ emission ratio results in significant over-estimates of those trends.

A much better tracer than CO or C_2_H_6_ to identify processes affecting atmospheric CH_4_ is the isotopic composition of CH_4_, which is emitted and removed by exactly the same processes as CH_4_ itself, though with isotope signatures that vary by source, and fractionation factors that are unique to various sink pathways. Here, we focus on *δ*^13^C_CH4_, which can be used to partition the relative fraction of emissions from broad categories: microbial (mic), fossil (fe; including natural fossil emissions)) and biomass burning (bb). Globally averaged atmospheric *δ*^13^C_CH4_ is equal to the average *δ*^13^C_CH4_ of sources mass-weighted by their emissions (equation (2.4)) and corrected for fractionation by sinks [23] (equation (2.5)).

2.4 $$\delta^{13}{\text{C}}_{\text{Q}}\cdot {\text{Q}}_{\text{Total}}=\delta^{13}{\text{C}}_{\text{mic}}\cdot {\text{Q}}_{\text{mic}}+\delta^{13}{\text{C}}_{\text{fe}}\cdot {\text{Q}}_{\text{fe}}+\delta^{13}{\text{C}}_{\text{bb}}\cdot {\text{Q}}_{\text{bb}}\text{, }$$

and, at steady-state,

2.5 $$\delta^{13}{\text{C}}_{\text{Q}}=(\varepsilon +1)\delta^{13}C_{\text{A}tm}+\varepsilon ,$$

where the subscript ‘Q’ refers to sources. An isotopic fractionation factor, *α*, is defined as the ratio of rate coefficients (k^13^C/k^12^C) for the reaction of ^13^CH_4_ relative to that for ^12^CH_4_ for a specific sink (e.g. reaction with OH). It is related to *ε* by *ε* = (*α *− 1) In equation (2.5), *ε* is weighted by the relative contributions of each sink process and commonly reported in per mille (‰). Interpretation of the isotope data requires knowledge of the mass-weighted isotopic composition of the sources that emit CH_4_ to the atmosphere. An isotope source signature database compiled by Sherwood *et al*. [24] greatly increased the sample size and reduced the uncertainty of source signatures for a global CH_4_ isotope mass balance. Within each source category, a range of *δ*^13^C_CH4_ values has been observed as a result of systematic spatial differences in these source signatures. These differences help constrain the spatial distribution of CH_4_ emissions. This database was complemented by other studies on the spatial patterns in *δ*^13^C_CH4_ of sources ([25] for oil, natural gas, coal, biomass burning and ruminants; [26] for wetlands; [27] for geological seeps; and [28] for ruminants), which can help to better leverage the spatial information in observed *δ*^13^C_CH4_._._Adjustment for fractionation by sinks, as with emissions, is weighted by the magnitude of each sink process. Since atmospheric observations of CH_4_ isotopes are sensitive to CH_4_ sources and sinks with their distinct fingerprints, they are an extremely powerful constraint on the CH_4_ budget (see the following section for the *δ*^13^C_CH4_ constraints on global CH_4_ budget).

**Figure 2.**
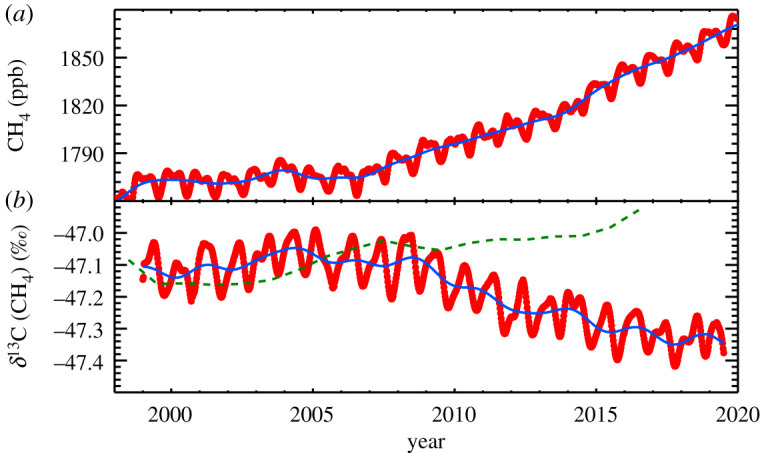
(*a*) The same as figure 1, but for 1998 through 2019. (*b*) Globally averaged *δ*^13^C(CH_4_) calculated from a subset of the samples used in (*a*). The dashed green line is from a model simulation where the OH component of *τ*CH_4_ is adjusted to drive the increase in atmospheric CH_4_ burden. (Online version in colour.)

In figure 2, CH_4_ abundance (2a) and *δ*^13^C_CH4_ (2b) are plotted. The start of the decline in *δ*^13^C_CH4_ is nearly coincident with the increase in atmospheric CH_4_ budget. Unfortunately, INSTAAR measurements of *δ*^13^C_CH4_ started after the large El Niño of 1997/1998, when there were large biomass burning signals, which *δ*^13^C_CH4_ should be very sensitive to. But based on analysis of measurements of CH_4_ abundance and *δ*^13^C_CH4_ at Ny Ålesund, Spitzbergen, Morimoto *et al*. [29] found significant enhancements of CH_4_ emissions from both wetlands and biomass burning in 1998, highlighting that the interpretation of the observations can be complicated by multiple processes changing nearly simultaneously. From measurements of air extracted from ice cores, we know that *δ*^13^C_CH4_ was increasing for approximately 200 yr [30] before the recent decline began. This suggests a rather significant change in the global budget of CH_4_ sources and sinks that will be explored below, although the timing of the change in emissions is difficult to match directly with the *δ*^13^C_CH4_ observations because of the slow response of *δ*^13^C_CH4_ to changes in CH_4_ emissions or average source signatures [31].

## 3. The global CH_4_ budget, past and present

Based on measurements of CH_4_ abundance from ice cores and firn, and assuming its lifetime was similar to today (approx. 9 yr), pre-industrial emissions were approximately 220 Tg CH_4_ yr^−1^. The dominant source then was emissions from wetlands, but there is substantial uncertainty with regard to emissions from natural fossil sources, which impacts estimates of anthropogenic fossil emissions in today's budget. *δ*^13^C_CH4_ can be used in an isotope mass balance to assess the fraction of total CH_4_ emissions from fossil sources [32], but it cannot distinguish between natural seeps and anthropogenic emissions from current fossil fuel exploitation.

A potential tracer for that is ^14^C_CH4_ in pre-industrial air extracted from ancient ice, because all ^14^C has decayed in fossil CH_4_ (lifetime approximately 8000 yr). While the number of observations of ^14^C_CH4_ in air extracted from ice cores remains very small (in large part because of the enormous amount of ice needed for sufficient air for a measurement), two studies now indicate that emissions from fossil sources prior to industrialization were small. Petrenko *et al*. [33] found emissions were less than approximately 15.4 Tg CH_4_ yr^−1^ based on ice with air age from 11.6 kyr ago. In a study representative of the atmosphere just prior to industrialization, Hmiel *et al*. [34] reported a fossil component of less than 5.4 Tg CH_4_ yr^−1^. These conclusions were based on very few ^14^C_CH4_ measurements, but if we assume natural fossil emissions have not changed since pre-industrial, then there is a large discrepancy between this direct quantification and aggregating assessments that suggest much greater natural geological fossil emissions with a range of 43–50 Tg CH_4_ yr^−1^ [27]. This large, important discrepancy will only be resolved with far more measurements of ^14^C_CH4_ from ancient pre-industrial ice complemented by systematic measurement of fluxes from seeps.

The main sources of CH_4_ to the atmosphere today are known, and its budget of emissions and sinks has been summarized periodically by the Global Carbon Project, most recently by Saunois *et al*. [35]. While these budget assessments use *in situ* observations and satellite retrievals of CH_4_, they do not apply the constraints imposed by measurements of CH_4_ isotopic composition.

Briefly, from comparison with a pre-industrial atmospheric CH_4_ mass balance assuming the same lifetime as today, we know approximately two-thirds of current CH_4_ emissions are anthropogenic and come from agriculture (ruminants and their manure, and rice agriculture), waste management (landfills and waste treatment), fossil fuel use (coal, oil and natural gas) and biomass burning (including biofuels). The remaining emissions are from natural processes, predominantly tropical and high-northern latitude wetlands. There remains a great disparity between bottom-up and top-down estimates of natural emissions [35]. If natural fossil emissions are indeed small, as Hmiel *et al*. [34] infer, then to balance the isotopic budget, anthropogenic fossil fuel emissions are correspondingly larger.

The largest atmospheric loss process in the global CH_4_ budget is mostly initiated by reaction with OH, especially in the tropical mid-troposphere [36], but also by Cl and O(^1^D) (stratosphere only). Oxidation by microbes in soils is likely a small sink, but uncertainty in its magnitude and trend remain large [37].

**Figure 3.**
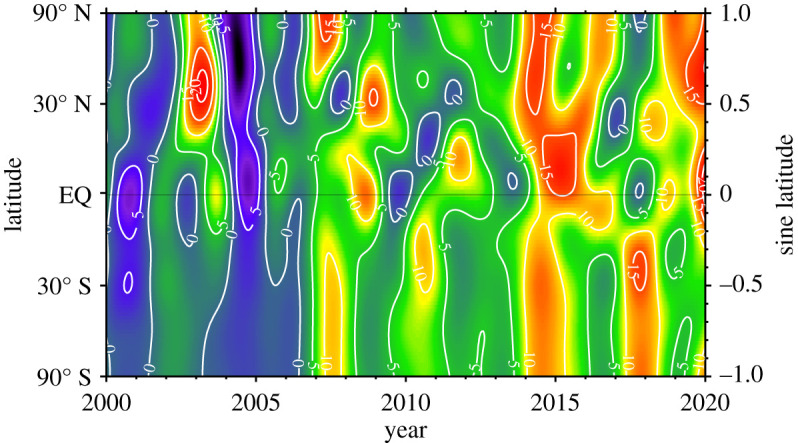
Contours of zonally averaged CH_4_ growth rate from 2000 through 2019. Use of sine latitude on the y-axis weights each latitude band by its atmospheric mass. (Online version in colour.)

Atmospheric observations of spatial gradients and their changes with time are used in atmospheric CTM to infer emissions from specific regions. In figure 3, contours of CH_4_ zonally averaged growth rate determined from NOAA's observations are plotted; warm colours show where the combined effects of source/sink imbalance and transport result in greater than zero growth rate and cool colours where it is less than zero. Data assimilation systems (also known as inverse modelling) start with an initial distribution of atmospheric CH_4_ and a first-guess distribution and magnitude of emissions. This first-guess is used to calculate atmospheric CH_4_ at the next model time step after winds and sinks have acted. Based on a comparison of the model and measured CH_4_, the emissions are optimized to get the best fit to the data. North-south gradients in growth rate, to first order, are qualitative indicators of where emissions have likely changed, but to be quantitative, the use of a CTM is absolutely necessary, except perhaps when global totals are estimated. This method has a limitation; it depends on the *a priori* first-guess for emissions. Optimized emissions can only be varied within the uncertainty limits specified for the first-guess, and they are also confined to the prior's spatial distribution. If that first-guess is grossly biased, the assimilation system cannot correct it enough, so emissions from another source are adjusted, perhaps erroneously, to match the observations. Improvements to bottom-up inventories and process models that supply priors would improve top-down assessments of the CH_4_ budget.

The renewed long-term increase in atmospheric CH_4_ and near-coincident decrease in *δ*^13^C_CH4_ in 2007 suggests a significant change in the global CH_4_ budget with potentially important implications for climate. Many scenarios have been proposed to explain the increase; Lan *et al*. [25] investigated some of them to determine which are consistent with observations of atmospheric CH_4_ abundance and *δ*^13^C_CH4_. Most scenarios look at a single process or combination of competing processes acting from 2007 to present, but the reality is likely more complicated than that.

Dlugokencky *et al*. [38] attributed the initial rise to increased CH_4_ emissions from tropical wetlands responding to increased precipitation in tropical land regions during an intense La Niña phase of ENSO in 2007 and 2008. But this change was not sufficient to sustain the increase; other factors, some perhaps that had acted over the previous decade must have contributed.

An isotope mass balance for CH_4_ requires that emissions from fossil sources (natural seeps and exploitation of fossil fuels) are a larger fraction of total emissions than captured in emission inventories [25,32,39], although with no significant trend [32]. Further studies using *δ*^13^C_CH4_ as a constraint on emission changes after 2006 are not consistent with increased fossil emissions dominating the increase in atmospheric CH_4_ burden [25,39]. A large increase in U.S. shale gas production contributing to the increase in global CH_4_ burden is not consistent with continental observations of CH_4_ abundance [22] nor with the heavy isotopic signature of U.S. shale gas [40].

Another possible explanation of rising methane is that [OH] has had a sustained decline, thereby increasing *τ*_CH4_. But this hypothesis is also inconsistent with the decade-long decreasing trend in *δ*^13^C_CH4_. Lan *et al*. [25] used a three-dimensional atmospheric transport model to investigate a scenario where the OH component of *τ*_CH4_ was adjusted to match the increase in CH_4_ burden. The impact on *δ*^13^C_CH4_ through 2016 is shown in figure 2*b* (green). As the proportion of oxidation by non-OH sinks becomes a larger fraction of the total sink, fractionation due to loss increases and the net effect is an increase in *δ*^13^C_CH4,_ opposite to what is observed. More generally, there is little evidence for a profoundly decreasing trend in [OH] (see e.g. [41]), but [OH], particularly its trend, remains a large uncertainty in quantifying the global CH_4_ budget and, especially, how it is changing.

Lan *et al*. [25] tested competing hypotheses explaining the post-2006 global CH_4_ increase in a common three-dimensional tracer model framework. The scenarios most consistent with both CH_4_ and *δ*^13^C_CH4_ observations and most plausible in regard to other evidence involve increased emissions from microbial sources (agriculture and wetlands) in the tropics with a small contribution from fossil sources [39,42–44], with a small decrease in biomass burning to maintain isotope mass balance.

## 4. Uncertainties that limit our understanding of the global CH_4_ budget

There are many processes for which our ability to assess IAV and trends is limited, and this imposes uncertainty in assessing atmospheric CH_4_'s global budget. Here, we highlight some of the larger uncertainties in our understanding.

### (a) Sink processes

Oxidation of atmospheric CH_4_ by OH is the largest term in the global CH_4_ budget, and small trends, much less than 1% yr^−1^, are important. We cannot accurately evaluate magnitudes and trends in global total CH_4_ emissions (nor determine which particular sources are changing), without an accurate understanding of the magnitude and trend in [OH]. To date, [OH] and its trend have been assessed indirectly through observations of 1,1,1-trichloroethane (CH_3_CCl_3_; common name: methyl chloroform, which we abbreviate to MC), an anthropogenic tracer predominantly removed from the atmosphere by reaction with OH. To be effective, the method required accurate estimates of MC emissions. When MC production was phased out by the Montreal Protocol on Substances that Deplete the Ozone Layer, it allowed Montzka *et al*. [45] to exploit the greatly decreased emissions of MC to estimate [OH] averaged over multiple years based on the rate of decay in the atmospheric burden of MC. However, as the abundance of MC in the atmosphere has decreased closer to its analytical detection limit, this approach has become more sensitive to small remaining emissions. Moreover, while this approach was useful for assessing the lifetimes of CH_4_ and MC averaged over some years and their IAV, it was less suitable to assess small trends in [OH].

Recent reports of a decreasing trend in [OH] driving the renewed growth in atmospheric CH_4_ since 2007 may be artefacts of the box-modelling methods used ([46] and references therein) and are inconsistent with models of atmospheric chemistry and transport [41]. This scenario is also inconsistent with trends in *δ*^13^C_CH4_ as discussed above. While the reaction rate coefficient for OH + CH_4_, including its temperature dependence, has been greatly studied [47], uncertainty in the fractionation of the reaction on atmospheric *δ*^13^C_CH4_ is still substantial, and that uncertainty affects our ability to quantitatively partition emissions among broad source categories. Saueressig *et al*. [48] reported a significantly smaller KIE for fractionation of ^13^C by OH (1.0039 ± 0.0004) than reported by Cantrell *et al*. [49] (1.0054 ± 0.0009). Uncertainty in this factor muddles interpretation of measurements of atmospheric *δ*^13^C_CH4_ imposing a 20% uncertainty on emissions from the fossil fuel sector (approx. 30 Tg CH_4_ yr^−1^) [25].

Oxidation of CH_4_ initiated by Cl has little impact on atmospheric CH_4_ total loss, but strongly fractionates *δ*^13^C_CH4_. Using a CTM (TOMCAT) and sources of Cl from natural and anthropogenic halocarbons and dechlorination of sea-salt aerosol, Hossaini *et al*. [50] estimated a sink for atmospheric CH_4_ by reaction with Cl of 12–13 Tg CH_4_ yr^−1^. Based on analysis of *δ*^13^C_CO_ measurements in the extra-tropical Southern Hemisphere, Gromov *et al*. [51] concluded that the loss of CH_4_ by reaction with Cl is no greater than 1% of the total atmospheric CH_4_ sink (less than half the estimate of Hossaini *et al*. [50]), and even 1% makes it difficult to balance the ^13^C(CO) budget. Wang *et al*. [52] calculated a similar size sink for CH_4_ from reaction with Cl, 5.3 Tg CH_4_ yr^−1^. The impact of Cl on the interpretation of *δ*^13^C_CH4_ observations is still clear; Strode *et al*. [53] compared multiple tropospheric Cl spatial and seasonal distributions and found the choice impacts the north-south gradient and seasonal cycle of *δ*^13^C_CH4_. And Lan *et al*. [25] found that the magnitude of the Cl sink chosen (none or 13 Tg CH_4_ yr^−1^) impacts the amount of fossil emissions that can be consistent with observations by approximately 20%.

Oxidation of CH_4_ in soils is intermediate in magnitude, much smaller than loss initiated by reaction with OH and likely much larger than reaction with Cl. Saunois *et al*. [35] reported a bottom-up range for soil loss of 12 to 49 Tg CH_4_ yr^−1^ based on a literature review. Fractionation of CH_4_ carbon isotopes by soil loss is reported to be approximately −20‰, with a range of measured values of −16 to 27‰ (e.g. [54]). Since the sink mostly occurs in forested soils, which are susceptible to land use, the magnitude [55] and distribution of the sink may be changing. Given that the fluxes of soil loss are very small and occur over large areas of forested and Savannah soils, better estimates of global soil loss will remain challenging.

### (b) Wetlands

CH_4_ emissions from wetlands are the largest natural source of atmospheric CH_4_. Emissions from wetlands vary greatly over small spatial scales and in time; this variability, combined with relatively few flux measurements, makes up-scaling uncertain. Prior estimates of emissions, therefore, rely on process models, but the range of emissions estimated among different wetland process models is quite large, approximately 40% [56]. One limitation is the accurate assessment of time-varying wetland area and inundation extent. While space-based methods seem ideal to determine wetland area, these methods are limited by cloud cover [57]. New methods based on the Cyclone Global Navigation Satellite System (CYGNSS), which operate in a frequency region that allows them to see through clouds, rain and most vegetation, may provide an important improvement [58]. Wetlands remain a limitation for top-down studies of the entire CH_4_ budget, because estimates of emissions from all other sources are impacted by inaccurate estimates of the magnitude and distribution of CH_4_ emissions from wetlands.

Using the isotopic constraint effectively requires that the source signatures of different wetland ecosystems are accounted for. Recently, Ganesan *et al*. [26] developed a map of spatially varying *δ*^13^C_CH4_ source signatures for wetlands, which vary by approximately 10‰ between high-northern latitudes and the tropics. The *δ*^13^C_CH4_ of CH_4_ emitted from a wetland will depend on factors such as the mechanism of CH_4_ production (CO_2_ versus acetate reduction), the source of the carbon (the proportion of plants with C3 versus C4 photosynthetic pathways), and oxidation between production and emission to the atmosphere, which depends on the transport route. Further efforts to identify the underlying processes that control wetland isotopic signature can improve the spatial mapping and thus improve the accuracy of attributing wetland emissions spatially.

## 5. Conclusion

Changes to the global CH_4_ budget are recorded in the atmosphere in CH_4_ abundance and *δ*^13^C_CH4_ spatial and temporal gradients. High-quality, calibrated observations of atmospheric CH_4_ and its stable isotopic composition are crucial for decreasing uncertainty in quantifying the evolving global CH_4_ budget, yet the world is still greatly under-sampled. If we want to track future changes in the global CH_4_ budget, including potential climate feedbacks, we need to expand these observations to under-sampled regions, such as the tropics, Siberia, Africa and South America. Most studies using CH_4_ isotope composition as a constraint use stable carbon isotopes, but more extensive observations of stable isotopes of hydrogen would further constrain the budget [39], especially for constraining the OH sink, which has a large fractionation factor for *δ*^2^H_CH4_. However, the use of *δ*^2^H_CH4_ would require both an extended time series from an adequate global network of air sampling sites and detailed work to define source signatures by type of source and geography.

Explaining the renewed and accelerating increase in atmospheric CH_4_ burden since 2007 remains challenging, and the exact causes are not yet clear. But, the observations we describe suggest that increased emissions from microbial sources are the strongest driver, with a relatively smaller contribution from other processes, e.g. fossil fuel exploitation. A more difficult question to answer is the one posed by this special issue: is warming feeding the warming? We cannot say for certain, but we cannot rule out the possibility that climate change is increasing CH_4_ emissions. The strong signals from the tropics combined with the isotopic data are consistent with increased emissions from natural wetlands, but large IAV and inter-decadal variability in wetland drivers like precipitation make it difficult to identify small trends. Observations are needed that will help process models capture this variability. The size of the IAV illustrates the potential scope of uncontrollable near-future change and emphasizes the urgency of reducing the global methane burden by mitigating the methane emissions that we can control, from the fossil fuel and agricultural sectors.

## Data accessibility

GML and INSTAAR data are available from NOAA's data server: ftp://aftp.cmdl.noaa.gov/data/trace_gases/xxx/flask/, where xxx = ‘ch4’ or ‘ch4c13’.
